# Preventing traditional management can cause grassland loss within 30 years in southern Brazil

**DOI:** 10.1038/s41598-020-57564-z

**Published:** 2020-01-21

**Authors:** Rafael Barbizan Sühs, Eduardo Luís Hettwer Giehl, Nivaldo Peroni

**Affiliations:** Department of Ecology and Zoology, Federal University of Santa Catarina (UFSC), Florianopolis, Santa Catarina Brazil

**Keywords:** Conservation biology, Ecosystem ecology, Fire ecology, Grassland ecology, Population dynamics

## Abstract

Woody encroachment threatens several ecosystems around the world. In general, management of grasslands includes regulation of fire and grazing regimes. Changes in these two types of disturbances are potential drivers of woody encroachment. Here we assessed how the traditional management carried out by local landholders affects a highland grassland ecosystem in southern Brazil. We hypothesized that grasslands converted to protected areas undergo fast woody encroachment. To reconstruct changes in vegetation, we interviewed former and current landholders and coupled their knowledge with an analysis of aerial and satellite images. During the first 11 years without fire and cattle, woody encroachment in grasslands increased exponentially. Woody encroachment occurred mostly by the replacement of grasslands by shrublands. Meanwhile, grasslands under traditional management remained almost unchanged for the last 40 years. The management of fire by local landholders has been part of their traditional practices for decades. Such management prevents large-scale wildfires and maintains natural highland grasslands. The quick pace of shrub encroachment in such grasslands threatens its exclusive diversity, human well-being and regional cultural heritage. Thus, conservation policies are needed to regulate and instruct about the use of fire as a management tool in highland grasslands of southern Brazil.

## Introduction

Extensive vegetation change can have important consequences in ecosystem functioning and economy^1^. For instance, woody encroachment (or woody plant encroachment), which is the increase in density, cover and biomass of shrubs or woody vegetation in grasslands^2^, has been reported around the world^3^. Woody encroachment is caused by several factors such as shifts in climate and biogeochemical cycles, changes in disturbance regimes (e.g. fire and grazing), or modification in ecological succession by introduction of non-native species or predator removal^2–5^. Woody encroachment alters fundamental ecological processes of ecosystems, including global carbon balances, reduction of water flow or groundwater recharge and the loss of biodiversity^4,6,7^. Because woody encroachment tends to negatively affect herbaceous vegetation, it constitutes a major threat to savanna and grassland ecosystems.

Control of woody encroachment is a key concern in rangelands. Woody encroachment reduces forage production, creates habitat for ectoparasites, and hampers animal handling^4^. Woody encroachment concerns land managers and methods for control and eradication include either fire, cutting trees, grazing, or a combination of these three methods^8,9^. Woody encroachment has been reported all over the world, *e.g*., southern Ethiopian savanna^8^, North American savanna, shrub‐steppe and grasslands ecosystems^10^, southeastern South American savanna^11^ (‘Campos’^6^), and Brazilian savanna (‘Cerrado’^12^). In highland grasslands of southern Brazil, extensive cattle grazing is part of the traditional management employed for centuries^13^. In addition, local ranchers control fire to promote sprouting of grassland vegetation and hinder forest expansion^6,14^. Fire use in the management of rangelands and protected areas is still controversial^15,16^, with an overlooking of fire and grazing by environmental agencies and protected area managers^12^. “Zero-fire” policies are adopted by several countries – including Brazil – to avoid and control fire in fire-prone ecosystems^15^. Despite some benefits of fire removal, such ecosystems face increasing risk of catastrophic fires because of the accumulation of flammable biomass. Catastrophic fires have detrimental effects on biodiversity^17^, human wellbeing and landscape cultural values^14^. When fire and grazing are prevented, tussock grasses and shrubs tend to replace small grasses and herbs^16,18,19^, leading unburned and ungrazed areas to changes in species richness and community composition^16^ and woody encroachment over time^6,17,20^. Although the fate of grasslands subjected to shrub encroachment might be difficult to predict^21^, shrub-encroached areas can either develop into forests (*e.g*.^22^) or remain in a stable shrubland state, where forest never develops^23^. Thus, the type of management used in rangelands can drive distinct vegetation dynamics.

High altitude grasslands ecosystems cover a wide geographical extent in southern Brazilian territory, delivering a huge number of benefits because of the ecosystem services they provide. Grasslands seem to be maintained by either fire or human activities, or both^24^ and are currently threatened by several factors, especially land use change^16^ and a weak set of conservation policies implemented by the Brazilian government^6^. In highlands, grasslands are interspersed with Araucaria forests (mixed rainforest)^24^, the latter being also threatened and reduced to ~12% of its original cover^25^. Mosaics containing Araucaria forests and highland grasslands integrate a domesticated landscape, which has been shaped by pre-Columbian societies via use and management of resources over millennia^13,26^. These societies contributed for a fast expansion of Araucaria forests since 1.5 ka BP. Furthermore, it is likely that pre-Columbian societies managed fire^26,27^, once it became frequent in that period^28^. Nowadays, fire is frequently used by local ranchers to promote grassland resprouting, which is beneficial for cattle grazing^19^. These activities (extensive cattle and burning) slow down natural forest expansion over grasslands^6,29^, contributing for the maintenance of high diversity in grasslands and across the landscapes^14^, sustaining ecosystem functions^30^.

We carried out a study in a highland grassland ecosystem in southern Brazil to assess which types of management local landholders use and the effects of their traditional management on subtropical highland grasslands. We hypothesize that vegetation dynamics is determined by the type of management. Specifically, grasslands are maintained by either fire or grazing, whereas protected areas should undergo woody encroachment because both types of disturbance are prevented. The encroachment of woody vegetation after traditional management abandonment is still poorly known to Brazilian highland grasslands (*e.g*.^20^), especially over different management types. Moreover, vegetation dynamics have never been evaluated between areas either with or without traditional management. We interviewed current and former landholders in order to understand which management practices they have been using in their lands. We then analyzed aerial photographs and satellite images to quantify vegetation changes. These two sources of information were used to check whether changes in vegetation depended on the type of management. We expected that traditional management would keep highlands grasslands by hindering woody encroachment, while areas without traditional management (no grazing and no fire) would allow for a fast woody encroachment.

## Results

### Interviews

We interviewed 36 landholders of 38 past or current rangelands. Five additional rangelands belonging to four more landholders were added because their property location and type of management were freely mentioned by our interviewees, especially by neighbors. Twenty-five properties have been managed with both cattle and fire, whereas 18 properties used to be managed in this way but are currently within the São Joaquim National Park (SJNP) protected area. Eleven rural properties were left out of our analysis because they did not meet our selection criteria, *i.e*., were located in areas with high geometric lens distortion (*i.e*. in the edges of aerial photographs – four properties), or had low grassland cover (less than 2 hectares – five properties), or no available image (two properties).

Together, all rangelands cover more than 12,000 ha (average ± standard deviation = 284.8 ± 341.6 ha) and are at ca 1500 m above sea level (1503.8 ± 146). The average time families own each property was 125.5 ± 78.5 years. Extensive cattle farming is the primary income, with an average density of 0.41 ± 0.13 animals ha^−1^. Fire was or is used by all landholders every ca 2 years (2 ± 0.5), in the end of the winter (August/September), to accelerate grassland regrowth for cattle. Lacking cattle and fire, 19.4% of landholders believe grasslands remain stable, 55.6% believe grasslands turn into shrublands, and 11.1% believe grasslands become forest over time. Table 1.

**Table 1:** Detail on the evaluated lands and management practices in subtropical highland grasslands in southern Brazil.

L ID	O ID	Elevation (m)	Land area (ha)	Period (years)	Cattle density (animals.ha^−^¹)	Fire frequency (years)	Grasslands’ fate	Grassland 1978 (ha)	Grassland 2018 (ha)	WE (%)	P	YWTM
1***	a	1571.4	1200	116	0.3	2	*“shrubland”*	368.8	367.6	0.41	No	0
2	b	1650.0	340	300	0.38	2	*“grassland*/*shrubland”*	146.4	145.9	0.38	No	0
3	c	1250.0	105	100	0.57	2	*“shrubland”*	23.6	23.6	0.38	No	0
4	d	1616.7	319	100	NA	2	*“shrubland*/*forest”*	8.5	5.0	35.18	Yes	8
5	e	1650.0	840	30	0.24	2	*“forest”*	25.8	22.4	14.60	Yes	11
6*	f	1650.0	58.4	80	0.37	2	*“shrubland”*	27.4	27.2	0.56	Yes	9
7	f	1650.0	68.5	80	0.37	2	*“shrubland”*	60.8	59.9	1.41	Yes	9
8	f	1625.0	114	80	0.37	2	*“shrubland”*	37.2	31.8	11.27	Yes	9
9	g	1650.0	17.4	130	0.57	3	*“shrubland”*	12.1	12.1	0.05	No	0
10	h	1650.0	68.8	200	0.5	2	*“forest”*	27.1	26.4	2.40	No	0
11	i	1650.0	65	120	0.46	4	*“grassland”*	17.5	17.5	−0.14	No	0
12	j	1650.0	65	100	0.42	2	*“grassland”*	19.6	19.5	0.43	No	0
13	k	1525.0	150	80	0.26	2	*“forest”*	11.5	11.2	2.14	No	0
14	l	1516.7	190	150	0.42	1	*“shrubland”*	28.1	11.7	63.60	Yes	9
15	m	1400.0	148	100	0.47	2	*“shrubland”*	45.6	43.2	3.18	No	0
16	n	1450.0	118	60	0.42	2	*“shrubland”*	8.3	8.3	−0.44	No	0
17	o	1416.7	800	241	0.37	2–3	*“grassland”*	353.2	322.2	0.15	No	0
18	p	1400.0	800	62	0.31	2	*“shrubland”*	451.7	448.8	0.03	No	0
19	q	1700.0	550	250	0.33	2	*“shrubland”*	146.3	145.2	0.41	No	0
20	r	1416.7	700	55	0.26	2–3	*“shrubland”*	330.5	330.7	0.06	No	0
21	s	1450.0	114	80	0.4	1	*“shrubland”*	19.4	12.6	27.34	Yes	9
22	s	1450.0	154	80	0.4	1	*“shrubland”*	9.1	1.1	76.74	Yes	9
23	t	1600.0	300	35	0.33	2	*“shrubland*/*forest”*	16.5	6.6	45.94	Yes	5
24	u	1300.0	155.8	150	0.51	2	*“shrubland”*	54.6	50.8	4.15	No	0
25	v	1650.0	1500	150	0.27	2	*“shrubland”*	380.8	162.8	54.70	Yes	8
26	v	1521.4	1000	150	0.2	2	*“shrubland”*	37.9	37.0	−0.76	No	0
27	w	1650.0	200	100	0.5	1	*“shrubland”*	91.9	87.8	4.31	No	0
28	x**	1550.0	169	NA	NA	NA	NA	109.2	104.7	4.51	No	6
29	x**	1450.0	300	NA	NA	NA	NA	132.5	103.6	21.17	Yes	10
30	y**	1450.0	135	NA	NA	NA	NA	42.9	39.6	7.43	No	6
31	z**	1650.0	101	NA	NA	NA	NA	56.5	45.2	19.12	No	9
32	aa**	1500.0	36.8	NA	NA	NA	NA	16.8	14.2	15.12	No	9
33	bb	1220	199	40	NA	2	*“grassland”*	NA	NA	NA	No	NA
34	cc	1070	218	56	0.28	2–3	*“shrubland”*	NA	NA	NA	No	NA
35	dd	1300	41.5	80	0.72	NA	NA	NA	NA	NA	No	NA
36	ee	1550	120	38	0.25	2	*“shrubland”*	NA	NA	NA	No	NA
37	ff	1600	260.5	29	0.69	2	*“forest”*	NA	NA	NA	Yes	NA
38	gg	1550	120	150	NA	2	NA	NA	NA	NA	Yes	NA
39	hh	1500	65	250	0.38	2	*“grassland”*	NA	NA	NA	Yes	NA
40	ii	1280	150	150	0.56	3	*“grassland”*	NA	NA	NA	No	NA
41	jj	1300	60	200	0.67	2–3	*“shrubland”*	NA	NA	NA	No	NA
42	kk	1540	67	300	0.52	2	*“grassland”*	NA	NA	NA	No	NA
43	ll	1445	62.5	300	0.48	NA	*“shrubland”*	NA	NA	NA	No	NA

L ID = land ID; O ID = Owner ID; Period = Period the property is within the owner/family; WE = Woody encroachment; P = Within protected area (cattle and fire are prevented); YWTM = number of years without traditional management. Grasslands’ fate: the main answer of interviewees for the question: “What happens to grasslands if fire and cattle are excluded?”. Grassland 1978: grassland area computed in polygons in 1978. Grassland 2018: grassland area computed in polygons in 2018. *property was removed from the analysis due to a recent fire in grasslands. **owners that were not interviewed. *** land ID 1 has three owners (all interviewed) who use the same management techniques. NA = Data not available.

### Imagery

From 1978 images, 73 polygons were drawn in grasslands of selected rangelands, covering an area of 3,191 ha. The extent of grasslands within polygons was 3,118.1 ha in 1978, decreasing 12% (2,746.2 ha) in 2018. The average time rangelands were kept since abandonment of traditional management (thus converted to protected areas) was 8.4 ± 1.6 years.

### Woody encroachment

**Figure 1:**
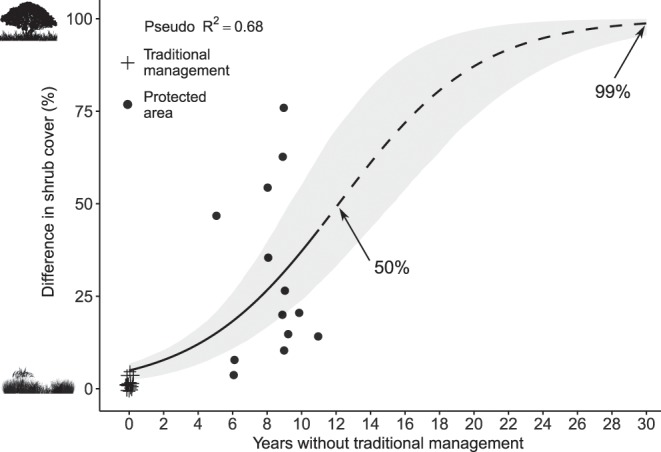
Effect of land management on shrub cover (calculated from the difference in shrub cover between the years 2018 and 1978) in southern Brazilian highlands. Solid line represents interpolation and dashed line represents extrapolation. Shaded areas represent 95% confidence intervals built from predictions based on 1000 bootstrap replicates of the original data. Arrows indicate 50 and 99% of shrub cover in grasslands (12 and 30 years, respectively).

Shrub encroachment accelerated with time since abandonment of traditional management (w_AIC_ = 0.73), being very little affected by elevation. Table 2. The model containing only time since abandonment of traditional management explained 68% of variation in shrub encroachment. Figure 1. In areas where traditional management still consists of using fire and grazing cattle, shrub encroachment remained around 1% year^−1^. Conversely, areas which became protected, thus excluding cattle and fire, experienced changes increasing exponentially over time, with an average shrub encroachment of 4.8% year^−1^ (ranging from 0.03 to 9.1% year^−1^). The time needed to shrubs to encroach into 50 and 99% of grasslands was estimated in 12 and 30 years, respectively. Field expeditions confirmed that the main shrub species encroaching in areas without traditional management is the shrub *Baccharis uncinella* DC. (Asteraceae).

**Table 2:** Set of produced models for evaluating shrub encroachment in relation to elevation and traditional management in southern Brazilian grasslands.

Int	Elev.	YWTM	Elev. × YWTM	df	logLik	AICc	ΔAIC	w_AIC_
−2.965	—	0.244	—	3	58.22	−109.52	0.00	0.73
−2.400	−0.0004	0.246	—	4	58.25	−106.90	2.62	0.20
−3.778	0.0005	0.767	−0.0003	5	58.67	−104.84	4.68	0.07
−1.801	—	—	—	2	46.35	−88.27	21.25	0.00

Int = Intercept; Elev. = Elevation; YWTM = years without traditional management, df = degrees of freedom, logLik = log-likelihood, AICc = Akaike information criteria corrected for small samples, wAIC = Akaike weight. Interaction between predictors is represented by “ × ”. Selected model follows a “*”. Models ordered by increasing values of AICc.

## Discussion

Changing land management had great impact in vegetation dynamics in our study system. Our results indicate that after cattle and fire withdrawal, grasslands underwent an exponential increase of shrub encroachment over time. Shrub encroachment proceeded in a fast pace in protected areas, replacing large extents of grasslands over short time. Following that pace, shrubs would entirely encroach over grasslands in ~30 years. Conversely, areas keeping traditional management faced little to no shrub encroachment over the last 40 years. These results corroborate our hypothesis and its consequences are discussed below.

Woody encroachment affects open-canopy ecosystems across the world, unleashing a need to understand its causes and consequences. Woody encroachment has been reported on African savannas^1,8^, North American savannas and grasslands^10,31,32^ and South American savannas and grasslands^6,12,16^, thus posing these ecosystems at risk. Several factors and their interactions cause woody encroachment, even though changes in disturbance regimes, such as fire and grazing, are amongst the most frequent drivers^3–5,21^. Therefore, the type of land management seems to be of paramount importance for landscape maintenance. For example, grasslands of subalpine ecosystems are being transformed into woodlands, facing shrub encroachment process as agricultural practices are abandoned^33^. In southern Brazil, grasslands converted into pine monocultures are vulnerable to shrub encroachment after pine removal, requiring active restoration^16^. In southeastern South America, the Uruguayan Savanna ecoregion faces a limited forest expansion because of cattle grazing and fire, although tree cover is favored by precipitation^11^. Our study supports the claim that highland grasslands in southern Brazil are prone to woody encroachment^6,20^, especially in the absence of fire and cattle grazing. Although substantial increases in woody encroachment can occur over decades^4^, we found evidence for an acceleration of shrub encroachment over time in areas where fire and cattle were excluded. Such management is normally enforced in protected grasslands (*e.g*.^16,30,34^). Meanwhile, native grasslands were maintained under traditional management by keeping large herbivores and fire. In North American drylands, for example, rates of encroachment vary from 0.1–2.3% year^−1^, depending on the ecoregion^4,10^. Here we found rates of shrub encroachment ranging from 0.03 to 9.1% year^−1^. In areas under traditional management, the average rate of shrub encroachment was 1.1% year^−1^, while in areas where traditional management was prevented the average rate was 4.8% year^−1^. Our results indicate a clear effect of the type of management on woody encroachment and highlights the fast pace it may follow.

Replacement of grasslands by woodlands has important consequences at both the community and ecosystem levels^2,32^. In general, chief consequences of woody encroachment are alteration of fundamental ecological processes and loss of biodiversity^3,6,7,32^. In addition, woody encroachment reduces the outcome of cattle production thus affecting human economies^4,35^. In the study region, all landholders have been using extensive cattle farming as their primary income for decades. In addition, all landholders manage fire to promote the regrowth of grasslands for cattle foraging in the end of the winter. Thus, it is not surprising that humans seem to shape ecosystems to reach their intended benefits. Over the last centuries, woody vegetation seems to be favored by the suppression of fire and grazing in subtropical ecosystems^17^ and the dependency of southern Brazilian highland grasslands on fire and grazing has been already reported (*e.g*.^6,16,20,24,36–38^). Fire and grazing are key elements of traditional management taken by local landholders in such system^13,14^. However, use and management of fire is still controversial^6,15^ and rarely described even in fire-managed pastoral systems^12^. Furthermore, Brazilian environmental agencies and protected area managers struggle to understand fire-managed pastoral systems^12^, which urges the creation of conservation policies that enable the use of fire in fire-prone ecosystems^15^. Fire brings benefits. For example, species richness of abandoned grasslands in southern Brazil can only be maintained by fire^6^. In Brazilian highland grasslands, fire and grazing exclusion can lead to decreases in species richness, changes in grassland physiognomy and species composition^16^, which can cause changes in ecosystem functioning^30^. In mesic grasslands of central United States, the prevention of woody encroachment by frequent burning is the best option^32^. In African moist and arid savannas, fire is an effective method for controlling shrub encroachment^9^. However, substantial ecosystem changes following woody encroachment may, in some cases, impair ecosystem recovery even when fire is reintroduced^32^. Also, fire must be used with responsibility, as in some cases it can favor invasive exotic species adapted to fire (*e.g*.^39,40^) or reach into forests when not adequately supervised or when the shrub component in the forest-grassland edge is too dense.

In general, few woody species tend to become aggressive encroachers or originate the encroachment process^41^. The main shrub species encroaching in the studied grasslands is *Baccharis uncinella*, which is a regionally common but endemic species to southern Brazilian highlands^42^. This species can expand from forest borders toward grasslands^20^ and facilitate the arrival and development of other shrubs and forest species, including nurse trees, potentially accelerating forest expansion^22,43^, as well as serving as a native fauna refugee^36,38^. A similar shrub-facilitation system has been reported in subalpine grasslands in Spain, in which a species of shrub that invaded grasslands facilitated the settlement and expansion of another shrub species^33^. We understand that a native shrubby vegetation, which seems to be a transient alternative state, can be beneficial at some level (see^44^) and deserves to be protected as well (see^36–38^). However, larger native grassland areas affected by woody encroachment as a result of fire and grazing suppression, can alter grassland communities, especially by reducing native forbs abundance and plant species richness^45^, as well as altering ecosystem functioning^30^. Besides, encroachment by *B. uncinella* seems to be facilitated by high air temperatures and native grasses^23^ and even monocultures^16^. At low to moderate densities, encroachment by *B. uncinella* seems to be easily controlled by fire. Under high density of *B. uncinella*, however, landscapes may be threatened by catastrophic fires.

Our findings show that highland grasslands that became protected by law – where both cattle grazing and fire are suppressed – faced woody encroachment. Conversely, highland grasslands under traditional management – with cattle grazing and fire – were maintained. Such management consists of 2-year fire interval, where different patches of grasslands are burned in each year, and low density of extensive grazing cattle (~0.41 animals ha^−1^). Most landholders we interviewed believe that, in the absence of cattle and fire, grasslands would turn into shrublands over time. Accumulation of flammable biomass, including that from *B. uncinella* encroachment, also concerns several of the landholders. In addition, landholders reported that unburned grasslands accumulate fuel and that shrubs facilitate fire spread to adjacent forests, because shrubs increase vertical reach of flames. Indeed, flammable biomass accumulates over time in grasslands areas where fire is prevented^16,17,19^ and shrubs, which are kept in low abundance in managed systems, establish and increase in abundance mostly from forest edges^20^. Once inside forests, fire can spread throughout the flammable litter of Araucaria trees. Araucaria is an abundant species and produces great part of the litter in Araucaria forests^46^. Also, litter in these forests have a lower decomposition rate compared to other forests (*e.g*., rainforests) because of the presence of oils and resin in Araucaria litter^46^, allowing fire to spread. Such characteristics may facilitate the occurrence of catastrophic fires, which impose a risk to biodiversity^17^, human wellbeing and cultural landscapes^14^. Natural grasslands, besides providing a more reliable carbon sink, can be more resilient to drought and wildfires than forests^47,48^. Therefore, policy makers and protected area managers should recognize fire as a natural and critical process in grasslands^15^, comprehend that degradation can affect protected grasslands^30^ and recognize that disturbances can be crucial to protect biodiversity-related and cultural aspects of the landscape.

## Conclusion

We found additional evidence for woody encroachment by shrubs in grasslands from southern Brazil highlands. Yet, the most outstanding fact of our results is the pace of vegetation change exponentially accelerating during the first 11 years. Consequently, large extents of grasslands can be replaced by shrubs after just a decade of management change. In addition, we believe that local landholders (ranchers) understand consequences of their management – which includes fire and grazing by cattle – on both the maintenance of grasslands and the prevention of large-scale destructive wildfires. Based on such knowledge and vegetation change analysis, we suggest that the quick pace of shrub encroachment in the region threatens not only grassland ecosystems and its unique biodiversity, but also human wellbeing and cultural heritage of the landscape. Such results highlight the need of grassland conservation policies allowing and instructing on the timing, frequency and extent of prescribed fire regimes as a management tool for both rangelands and protected areas.

## Methods

### Study area

**Figure 2:**
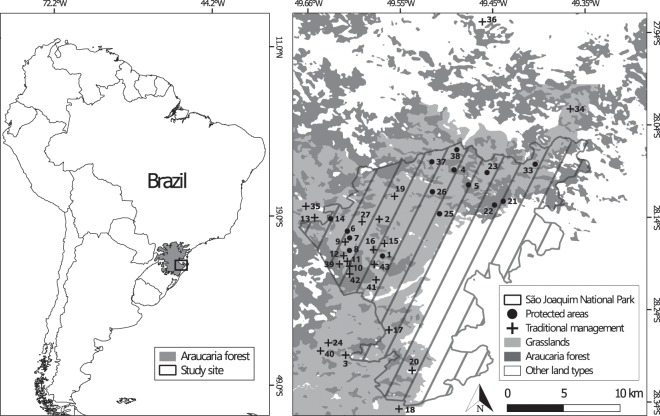
Location where the study was developed and the evaluated properties in southern Brazilian highlands. More details on evaluated properties can be found on Table 1. Map created with the software QGis platform, version 2.18.20^52^.

The study was conducted in the highlands of southern Brazil, in protected areas and rangelands in the São Joaquim National Park (SJNP) region, state of Santa Catarina, Brazil. Figure 2. This region encompasses one of the highest elevated zones of southern Brazil, reaching up to 1800 m a.s.l. The protected area has 49,300 ha, of which 13,000 ha have been acquired from former landholders by the Brazilian government since 2006. The main ecoregions in these highlands are high-altitude grasslands and mixed rainforest (Araucaria forest). The climate between 1961 and 2016, recorded at the nearest weather station (distant ca. 30 km), was characterized by an annual mean rainfall of 1,626.3 mm.yr^−1^, equally distributed throughout the year, and an annual mean temperature of 13.3 °C. The average minimum temperature for the coldest month (July) was 6.0 °C and the average maximum temperature for the hottest month (January) was 22.9 °C. The minimum absolute temperature recorded was −9.0 °C and the maximum absolute temperature was 31.4 °C. During winter, frosts are common, and snows are occasional. Climate data compiled from^49^.

### Data collection

#### Interviews

In 2016 and 2017, we interviewed owners of existing rangelands and previous owners whose areas were acquired to join the SJNP. The participants were selected from a list of former and current landholders of the SJNP region provided by the SJNP manager. We tried to reach all landholders mentioned in the list. In addition to the provided list, we also employed snowball sampling to increase the number of participants. Before starting the interviews, we asked the participants whether they wanted or not to answer our questionnaire and a consent form with the study aims and legal consequences was delivered. This research followed the guidelines of the Ethics Committee from Federal University of Santa Catarina (CEPSH), which also approved the development of this research (CEPSH - 44039415.6.0000.0121; Number 1.095.964 of 08/06/2015). Statement on written informed consent has been obtained from all the participants. Questionnaires contained open-ended questions and followed a semi-structured guide. The questions aimed to get information on the property location and size, the residence and within-family ownership time, the type and frequency of management used in the area and their opinion on the fate of grasslands in the absence of cattle and fire. When cattle grazing was mentioned, we additionally asked how many animals they had. We asked the landholders to locate and delimit their current or past properties on a digital map. The authorization for developing this study in the protected area and private properties was approved by the Brazilian government (SISBio code 48898–1) and the landholders, respectively.

#### Imagery

**Figure 3:**
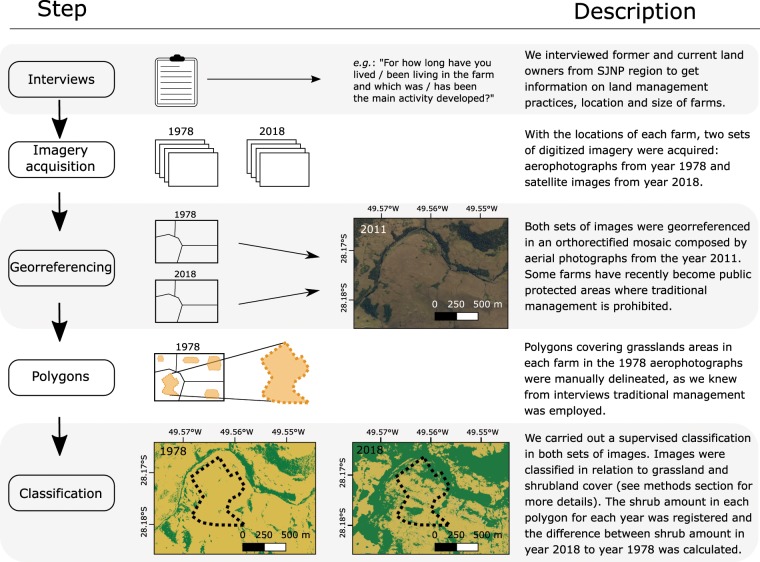
Schematic representation of each step taken before analysis, from interviews to image classification in southern Brazilian highlands. Example figure taken from freely available imagery from Google Earth. Image classification was carried out in MultiSpec software, version 3.4^51^ (https://engineering.purdue.edu/~biehl/MultiSpec/). QGis platform version 2.18.20^52^ was used for georeferencing, mosaic building and area measurements.

Acquisition and georeferencing. Aerial photographs taken in 1978 were acquired from a local public organ (Secretaria de Planejamento do Estado de Santa Catarina). These images have ~5490 × 5575 pixels (width × height) and 600 pixels inch^−1^ resolution. Very high-resolution satellite imagery (CNES’ Pleiades-1A data and Airbus’ SPOT series satellite data) taken in 2018 were acquired from Google Earth (GE - http://earth.google.com). Both 1978 and 2018 images were georeferenced in an orthorectified mosaic composed by aerial photographs from 2011 (Aerial Survey of the State of Santa Catarina – http://sigsc.sds.sc.gov.br). We used the Thin Plate Spline correction for both 1978 and 2018 images. The 2011 orthorectified mosaic was used as reference for ground control points (GCP). At least 20 GCP were established in each image, especially using roads, buildings and rocky outcrops as references. Figure 3.

Sampling procedure. We delimited polygons in which we quantified changes from past to current vegetation cover. Polygons were manually drawn for grasslands (on 1978 images) intersecting with past rangeland delimitation provided by interviewed landholders (Fig. 2). Forests were avoided whenever possible once our primary interest was to detect changes in grasslands and because preliminary inspection showed little changes in forest extent. Polygons were drawn at least 20 m away from buildings and from the edge of continuous forests, because such areas tend to rapidly change after traditional management abandonment^20^. We also placed polygons 20 m away from property limits, to avoid influence from neighbor areas for which we had no management information. Rangelands located in areas with high geometric lens distortion (*i.e*. in the edges of aerial photographs), with low grassland cover (*i.e*. less than 2 hectares) or with no available image, were discarded from the analysis.

Classification. We carried out a supervised classification using maximum likelihood algorithm. Because polygons were drawn for 1978 images, when traditional management was in place (data acquired from interviews), such images were classified in two classes: grassland and forest. In turn, 2018 images were classified in the following classes: grassland, shrubland, water bodies, rocky outcrops, plantations and buildings. Figure 3. Classification accuracy was assessed via Cohen’s coefficient (κ). We only accepted classifications when κ > 0.8, indicating an overall good performance^50^. Then, the area occupied by each class was calculated for each polygon. Finally, classes only observed in 2018 images (*e.g*. rock outcrops, water bodies, buildings and plantations) were discarded from the total polygon area in both times. We validated the classification outcome by field expeditions. Image classification was carried out in MultiSpec software, version 3.4^51^ (https://engineering.purdue.edu/~biehl/MultiSpec/). QGis platform version 2.18.20^52^ was used for georeferencing, mosaic building and area measurements.

### Data analysis

Because polygons varied in size depending on property settings, we obtained the proportional extent of every class over the whole polygon extent. We used beta regression models (with a logit link function) to model shrub encroachment, *i.e*. the increase in shrub area (%) from 1978 to 2018. Beta regression models are suitable to model proportions, because the beta distribution assumes values in the interval (0, 1)^53^. As predictors of shrub encroachment, we used the time since abandonment of traditional management (in years) and elevation. The interaction between both predictors was also tested, because we expected upper areas could change at a slower pace than lower ones within the same time extent. Model selection was based on the Akaike information criterion corrected for small sample sizes (AICc)^54^ and validated by a graphical analysis of residuals. We constructed 95% confidence intervals for the predicted values based on 1000 bootstrap samples from the original data^55^. All analyses were carried out in the R environment^56^. Package “betareg”^53^ was used for model building and predictions and “ggplot2”^57^ for visualization of model outputs.

## Data Availability

All data generated during this study are included in this published article (Table 1 – Results section).
